# Standard reference values of the upper body posture in healthy male adults aged between 41 and 50 years in Germany

**DOI:** 10.1038/s41598-020-60813-w

**Published:** 2020-03-02

**Authors:** Daniela Ohlendorf, Ali Gerez, Laurin Porsch, Fabian Holzgreve, Laura Maltry, Hanns Ackermann, David A. Groneberg

**Affiliations:** 1 Institute of Occupational Medicine, Social Medicine and Environmental Medicine, Goethe-University Frankfurt/Main, Theodor-Stern-Kai 7, Building 9A, 60590 Frankfurt/Main, Germany; 2 Institute of Biostatistics and Mathematical Modeling, Goethe-University, Frankfurt/Main, Theodor-Stern-Kai 7, Building 11A, 60596 Frankfurt/Main, Germany

**Keywords:** Anatomy, Skeleton

## Abstract

Background: Classifications of posture deviations are possible when they can be compared to the standard values for healthy persons. Standard values for healthy male adults aged between 41 and 50 years are currently missing. Methods: 100 healthy volunteers (41–50 years old; 45.37 ± 3.06 years) were included in the study. Their body weight ranged from 68 to 132 kg (88.76 ± 15.93 kg), their heights from 1.64 to 2.0 m (1.81 ± 0.07 m) and the Body Mass Index (BMI) ranged from 19.0 kg/m² to 37.7 kg/m² (26.2 ± 3.96 kg/m²). A three-dimensional back scan was performed to quantify the upper back posture during habitual standing. The upper and lower limit for 95% of the tolerance regions and the left and right limit of the confidence interval were calculated. Results: The upper body posture of the subjects was close to the symmetry, or 0°, axis. There was a moderate ventral upper body inclination with a slight left lateral axial deviation and rotation of the spine to the right. An enhanced kyphotic posture was observed in the sagittal plane in the area of the thoracic spine. The shoulder and pelvis areas were almost balanced. Conclusion: Healthy males between 41 and 50 years were found to have an almost balanced posture with minimal ventral body inclination and a marginal scoliotic deviation. These values allow a comparison with other studies for control and patient data and may serve as basis in both clinical practice and scientific studies.

## Introduction

The professional and social demands of the 21st century necessitate an increasing level of performance from every individual in highly developed industrial countries. The effects of such a lifestyle on the musculoskeletal system can be observed and the development of postural deformities and back pain intensifies. The general lack of movement and the resulting muscle insufficiency additionally increased the danger of pathological conditions of the musculoskeletal system^1–3^. In addition to prophylactic measures, such as regular physical activity, precise diagnostic aids are indispensable for an adequate therapy.

However, not only is the diagnosis of postural deformities of clinical interest, but also the basic question of an accepted physiological posture. To date, there are no scientifically clearly defined standard values for this which are both age- and gender-related^4,5^. Such representative norm values could be of great importance for a systematic diagnosis of postural deformities and allow the formulation of criteria for the classification of pathological and physiological conditions. Therapeutic decisions could be simplified. Although the primary aim of any therapy for postural deformities is first and foremost to alleviate the clinical symptoms of postural deformities, ideally it would also be desirable to achieve a physiological posture in order to stabilize the success of the therapy.

In everyday clinical life, there are various procedures for systematic clinical diagnostics to determine possible pathologies in the musculoskeletal system^6^. These primarily include clinical inspection and manual diagnostics by the orthopedic surgeon^7^. If, however, further information is necessary for therapy planning, the radiographic imaging examination is considered the gold standard despite its harmful side effects^8,9^. Due to radiation protection, however, the regular use of X-rays must be critically questioned with regard to low-risk initial diagnostics and possible follow-up after therapeutic measures^10^. In order to enable a radiation-free and, thus, harmless examination, it is now possible to use video raster stereography as an alternative method to X-rays^11–13^. It has been proven that the upper body posture and postural disturbances can be sufficiently recorded and evaluated with the help of this light-optometrical technique^14,15^. Thus, video raster stereography is considered today as a valid alternative method for the presentation of posture parameters and anomalies^16–19^.

In order to be able to make a scientifically recognized statement about which values for the postural parameters are to be regarded as the norm, representative studies with a sufficiently large homogeneous group of test persons must be carried out. To this end, using the technique of video raster stereography, studies^20,21^ have already included subject populations of 102 healthy men aged 18–35 years^20^ and 106 healthy women aged 21–30 years^22^. For middle-aged groups over 35 years of age, no comparable unisexual study to formulate representative norm values with regard to upper body statics has been carried out to date. Thus, standard values of men between 41–50 years are also missing.

Therefore, the aim of this study is to obtain these norm values from 100 healthy men aged between 41 and 50. so that they can be used in everyday medical practice for diagnosis and therapy.

## Material and Methods

### Subjects

**Figure 1:**
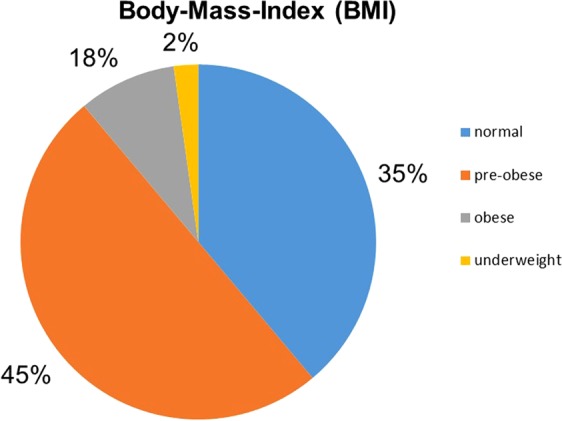
Frequency distribution of BMI according to WHO classification.

A total of 100 healthy, adult volunteers aged between 41 and 50 years (45.37 ± 3.06 years) participated in this study. The body weight ranged between 68 and 132 kg (88.76 ± 15.93 kg), the height between 164 cm and 200 cm (180.62 ± 7.29 cm) and the BMI between 19 and 37.3 kg/m² (26.02 ± 3.96 kg/m^2^). According to the World Health Organization (WHO) classification^23^, 35 subjects were classified as normal weight (BMI 18.5–24.9 kg/m^2^), 45 as overweight or pre-obese (BMI 25.0–29.9 kg/m^2^) and 18 as obese (BMI ≥ 30.0 kg/m^2^). A total of 2 of the 100 participants (2%) were underweight (BMI < 18.5 kg/m^2^) (Fig. 1).

All subjects were subjectively healthy without previous postural diseases and/or temporo-mandibular disorders. Each participant had to sign a written consent for voluntary participation and complete a medical history form before the start of the study. The anamnesis questionnaire of the Centre for Dental, Oral and Maxillofacial Medicine of the Goethe University Frankfurt am Main was used^24^. This initially included questions on general diseases such as osteoporosis or diabetes mellitus. Furthermore, it was asked whether they had pain in the joints, in general, as well as noises in the ears and complaints in the temporomandibular joint. The test persons were also asked to provide information about possible accidents in the mouth, jaw and face areas and in the musculoskeletal system. The subjects were also asked to indicate whether they had ever undergone orthodontic treatment, what their profession was and whether they were active in sports. Finally, body height and weight were recorded in order to assess the BMI. The evaluation revealed that the majority of the participants were employed in an office (58%), while 38% of the test persons were physically active in the context of their professional life although 4% of the participants did not wish to give any information about their occupation. In addition, around 45% of the selected participants regularly engaged in sports in their leisure time.

The study was in accordance with the 1964 Helsinki Declaration and its later amendments and was approved by the local medical ethics committee of the Faculty of Medical Science, Goethe University Frankfurt, Germany (approval No. 303/16).

### Measurement system

For the representation of the three-dimensional upper body statics, the contactless, light-optical back scanner “ABW-BodyMapper” of the company ABW GmbH (Frickenhausen/Germany) was used, the procedure of which is based on video raster stereography. The depth resolution of the generated resultant image is 1/100 mm and the maximum image frequency is 50 frames/sec. According to the manufacturer, measurement errors during recording should be <1 mm. For repeat measurements, this measurement accuracy was specified as less than 0.5 mm. In order to achieve optimum measurement of the back surface, for each test person six anatomical fixed points were provided, marked with a total of six self-adhesive, light-reflecting markers of 1 cm diameter. The ABW-BodyMapper back scanner has already been used successfully in studies to determine representative standard values for young men (18–35 years)^20^ and women (21–30 years)^22^.

### Measure protocol

The test persons stood barefoot in a habitual posture, approximately 90 cm in front of the back scanner, with their arms hung loosely, looking horizontally at the opposite wall. In order to obtain reproducible values, three repeated measurements were performed within 2 minutes.

### Evaluation of parameters

The values determined were divided into three categories according to the anatomical topography. The markers of the spinal column parameters ranged from the 7th cervical vertebra to the rima ani, while the shoulder parameters enclosed markers on the shoulder blade. The pelvic parameters were derived from the marker positions on the lumbar pits (posterior superior iliac spine). The precise placement of the six landmarks and also the definition of all calculated evaluation parameters is illustrated in the study protocol of Ohlendorf *et al*.^25^. The definitions of the parameters are specified and defined accordingly by the manufacturer and are therefore adopted in the following evaluations.

### Data analysis

Table 1 contains all the formulas for calculating each evaluation parameter.

**Table 1:** Formula or algorithm used to calculate the evaluation parameters.

Type of feature	algorithm used	formula	used for features
distance of two points in 3d space	euclidian distance	$d=\sqrt{{(Ax-Bx)}^{2}+{(Ay-By)}^{2}+{(Az-Bz)}^{2}}$	TrunkLengthD TrunkLengthS DimpleDistance AISDistance
difference in height	difference	d = Ax − Bx	PelvicPositionMM ScapulaePosition
perpendicular angle (sagittal)	trigonometry	angle = α tan $\frac{{\rm{Az}}-{\rm{Bz}}}{Ax-Bx}$	TrunkInclination
perpendicular angle (sagittal)	trigonometry	angle = α tan $\frac{{\rm{Ay}}-{\rm{By}}}{Ax-Bx}$	TrunkImbalance
horizontal angle	trigonometry	angle = α tan $\frac{{\rm{Ax}}-{\rm{Bx}}}{Ay-By}$	PelvicTorsion ShoulderPositionLeft ShoulderPositionRight
tranversal angle	trigonometry	angle = α tan $\frac{{\rm{Az}}-{\rm{Bz}}}{Ay-By}$	PelvicRotation ScapulaeRotation
angle to perpendicular (not in plane)	trigonometry	$angle=a\,\cos \,\frac{\overrightarrow{A}\ast \overrightarrow{B}}{|\overrightarrow{A}\ast \overrightarrow{B}|}$	ThoracicAngle LumbarAngle
statistical root mean square	statistics	$rms=\sqrt{\frac{1}{n}\mathop{\sum }\limits_{i=1}^{n}{x}_{i}^{2}}$	RMSLateralDeviation RMSRotation
maximal value	statistics	$m={{\rm{\max }}}_{i=1}^{n}|{x}_{i}|$	MaxlateralDeviation MaxRotation
angle in 3d space	trigonometry	$angle=a\,\cos ({(\overrightarrow{A}-\overrightarrow{B})}^{\circ }\ast {(\overrightarrow{C}-\overrightarrow{B})}^{\circ })$	TrunkAxisError KyphoticAngle LordoticAngle

### Statistical evaluation

The collected data were evaluated with the help of the software program BIAS (11.03) (Epsilon Verlag, Darmstadt/Germany)^26^. A possible normal distribution of the data was checked by the Kolmogoroff-Smirnoff-Test. Parametric or nonparametric tolerance ranges (TR) were calculated as defined by the lower and upper limits for 95% of all values (±2 SD values). These values have findings found in about 95% of the subjects. Values within this range were classified as “normal”. In addition, the confidence interval (CI) was calculated, which, depending on the distribution quality, indicates the range of the mean or median values and, thus, represents a measure of the accuracy of these values.

For correlations, the Spearman & Kendall rank correlation test was used for non-normally distributed data. The evaluation of the Evans correlation coefficient rho is defined as follows: rho < 0.2 poor, rho = 0.2–0.4 moderate, rho = 0.6–0.8 strong, rho > 0.8 optimum. The significance level for all tests was set to a p-value of 0.05.

### Ethics approval and consent to participate

This study was approved by the Ethics Committee (303/16) of the Goethe University Frankfurt am Main. All participants signed an informed consent to participate in advance. All participants signed an informed consent to participate in advance.

### Consent to publish

All individuals have given their consent to publish their images.

## Results

Of the constitutional parameters (Table 2), only body height was normally distributed, while body weight and BMI were not normally distributed. For height, the mean was determined to be 180.67 cm, with the TR having a lower limit of 166.05 cm and an upper limit of 195.29 cm. The CI had a left limit of 179.22 cm and a right limit of 182.12 cm. The subjects were found to have a median body weight of 85.0 kg (TR lower limit: 68.0 kg/upper limit: 130.42 kg; CI left limit: 82.0 kg/right limit: 89.0 kg). The BMI had a median value of 26 kg/m², whereby the lower limit of the TR was 20.55 kg/m² and the upper limit 37.15 kg/m². The CI had a left limit of 25.0 kg/m² and a right limit of 27.0 kg/m².

**Table 2:** Presentation of medians/averages, tolerance range and confidence interval with associated lower and upper, left and right limits in relation to height, body weight and BMI.

Parameter	Mean value	Median value	Tolerance range		Confidence interval	
			lower limit	upper limit	left limit	right limit
Body height (cm)	180.67	—	166.05	195.29	179.22	182.12
Body weight (kg)	—	85.0	68.0	130.42	82.0	89.0
BMI (kg/m^2^)	—	26.0	20.55	37.15	25.0	27.0

The data of the upper body posture are shown in Table 3. The average trunk length D of the subjects was 500.17 mm (TR lower limit: 443.47 mm/upper limit: 556.87 mm; CI left limit: 494.53 mm/right limit: 505.81 mm) and the trunk length S was 543.76 mm (TR lower limit: 479.42 mm/upper limit: 608.1 mm; CI left limit: 537.36 mm/right limit: 550.16 mm). The test persons were inclined 3.4° ventrally in the sagittal trunk decline. The lower limit of the TR was −8.47° and the upper limit 1.66°, while the left limit of the CI was −3.9° and the right limit −2.89°. Furthermore, there was a slight deviation to the left by 0.3°. The TR displayed both a left (CI lower limit at −3.01°) and a right lateral (CI upper limit at 2.39°) deviation in the frontal plane. The CI lay between −0.57° (left limit) and −0.03° (right limit), where both values displayed a left-lateral deviation. An axial decline (inclination of the upper body with respect to the horizontal between the right and left pelvis) was also minimal to the left with a value of <1° (0.83°) (TR lower limit: −5.78°/upper limit: 4.11°; CI left limit: −1.32°/right limit: −0.34°).

**Table 3:** Spine, shoulder and pelvis parameters: mean values, medians, tolerance region (upper and lower limits) and confidence interval (left and right limits).

	Mean value	Median value	Tolerance range lower limit	Tolerance range upper limit	Confidence interval left limit	Confidence interval right limit
**Spine parameter**						
*Trunk length D (mm)*	*500.17*	—	*443.47*	*556.87*	*494.53*	*505.81*
*Trunk length S (mm)*	*543.76*	—	*479.42*	*608.1*	*537.36*	*550.16*
*Sagittal trunk decline (°)*	*−3.4*	—	*−8.47*	*1.66*	*−3.9*	*−2.89*
*Frontal trunk decline (°)*	*−0.3*	—	*−3.01*	*2.39*	*−0.57*	*−0.03*
*Axis decline (°)*	*−0.83*	—	*−5.78*	*4.11*	*−1.32*	*−0.34*
*Thoracic bending angle (°)*	*15.76*	—	*8.56*	*22.96*	*15.03*	*16.48*
*Lumbar bending angle (°)*	*10.34*	—	*4.92*	*15.76*	*9.8*	*10.88*
Standard deviation lateral deviation (mm)	—	3.5	1.15	8.85	3.01	4.20
Maximal lateral deviation (mm)	—	−4.88	−13.88	8.91	−5.99	−2.99
Standard deviation rotation (°)	—	3.71	1.08	8.54	3.32	4.33
Maximal rotation (°)	—	3.78	−13.82	14.1	−2.7	6.0
Kyphosis angle (°)	51.08	—	31.63	70.53	49.14	53.01
Lordosis angle (°)	32.86	—	15.25	50.47	31.11	34.62
**Shoulder parameter**						
*Scapular distance (mm)*	*186.04*	—	*142.29*	*229.8*	*181.69*	*190.4*
*Scapular height (°)*	*−0.89*	—	*−12.37*	*10.59*	*−2.1*	*0.32*
*Scapular rotation (°)*	*1.65*	—	*−4.21*	*7.53*	*1.07*	*2.24*
Scapular angle left (°)	—	26.74	16.99	57.26	25.35	29.35
Scapula angle right (°)	—	28.49	12.82	45.54	27.59	29.94
**Pelvis parameter**						
*Pelvis distance (mm)*	*92.84*	—	*69.03*	*116.66*	*90.47*	*95.21*
*Pelvis height (°)*	*−0.5*	—	*−4.52*	*3.51*	*−0.9*	*−0.1*
Pelvis height (mm)	—	−1.13	−7.82	7.05	−1.48	−0.39
*Pelvis torsion (°)*	*0.12*	—	*−10.32*	*10.57*	*−0.92*	*1.17*
*Pelvis rotation (°)*	*0.19*		*−6.63*	*7.02*	*−0.48*	*0.87*

Parameters that are not displayed in italics are normally distributed, so that the mean is displayed. Non-normally distributed data are displayed in italics and show the median.

The thoracic and lumbar bending angles had median values of 15.76° and 10.34°, respectively. The TR for the thoracic bending angle ranged from 8.56° (upper limit) to 22.96° (lower limit), while the CI was between 15.03° (left limit) and 16.48° (right limit), respectively. Comparable TRs (upper limit 4.92°/lower limit 15.76°) and CIs (left limit 9.8°/right limit 10.88°) are available for the lumbar bending angle. The standard deviation lateral deviation had a right-sided inclination (3.5 mm) much like the TR (upper limit 1.15 mm/lower limit 8.85 mm) and the CI (left limit 3.01 mm/right limit 4.2 mm). The value for the standard deviation rotation was 3.71° indicating a right-sided rotation of the spine. The TR (upper limit 1.08°/lower limit 8.54°) and the CI (left limit 3.32°/right limit 4.33°) were congruent with this finding. The kyphosis angle was 51.08° with a TR of 31.63° (upper limit) and 70.53° (lower limit) and a CI of 49.14° (left limit) and 53.01° (right limit). The lordosis angle had a value of 32.86° (TR lower limit: 15.25°/upper limit: 50.47°; CI left limit: 31.11°/right limit: 34.62°).

In terms of shoulder parameters, the average shoulder blade distance was found to be 186.04 mm (TR lower limit 142.29 mm/upper limit 229.8 mm; CI left limit 181.69 mm/right limit 190.40 mm). With a value of <1 mm (0.89 mm), the left shoulder was more cranial than the right, with the TR taking a value of −12.37 mm (left shoulder more cranial) for the lower limit and 10.59 mm (right shoulder more cranial) for the upper limit. The CI was −2.1 mm with the left limit and 0.32 mm with the right limit. The mean value of the shoulder blade rotation shows, with a value of 1.65°, that the right shoulder was dorsally rotated. When looking at the TR, it is noticeable that its lower limit is −4.21° (left shoulder rotated dorsally) and its upper limit 7.53° (right shoulder rotated dorsally). The CI had a left limit of 1.07° and a right limit of 2.24°. When looking at the shoulder angles to the right and left, the right shoulder was found to be about 1.8 mm more caudal.

The average pelvic distance of the subjects was 92.84 mm, with the TR having a lower limit of 69.03 mm and an upper limit of 116.66 mm, while the left limit of the CI was 90.47 mm and the right limit 95.21 mm. Overall, the left pelvis was 0.5° and 1.13 mm higher than the right pelvis due to the pelvis heights, respectively. In addition, the right pelvic region was found to be minimally cranial and dorsally rotated compared to the left pelvis. For pelvis torsion, the TR had a lower limit of −10.32° and an upper limit of 10.57°, while the CI had a left limit of −0.92° and a right limit of 1.17°. The TR for the pool rotation parameter displayed a lower limit of −6.63° and an upper limit of 7.02°, while the left limit of the CI was −0.48° and the right limit 0.87°.

Table 4 shows the correlations between trunk length D and S and body weight, height and BMI. The difference between the two parameters lies in the length of the spine due to anatomical marker reference points. While the trunk length D is constituted by the distance between the marker on vertebra prominens and the center of the SIPS markers at spinal height, the trunk length S is the spatial distance between the markers on vertebra prominens and sacrum point. Both trunk lengths show no significant correlation to body weight. However, there is a significant correlation (p < 0.001 and 0.01, respectively) for both body height and BMI, with a positive correlation (0.58 and 0.63, moderate and strong effect size, respectively) for body height and a negative correlation (−0.25 and 0.29, moderate effect size, respectively) for BMI. This means that the larger the subjects, the longer the trunk length, but the longer the trunk length, the lower the BMI.

**Table 4:** Correlation (p-value and rho) of the parameters size, weight and BMI to trunk length.

	Body weight		Body heights		BMI	
	p-value	rho	p-value	rho	p-value	rho
Trunk length D (mm)	*0,57*	*0,06*	***0.0001***	*0,58*	*0,01*	*−0,25*
Trunk length S (mm)	*0,72*	*0,04*	***0.0001***	*0,63*	***0.001***	*−0,29*

Significant p-values are marked in bold. The trunk length D is defined as spatial distance between the markers VP and DM Spatial distance between the markers VP and SP. Italic data are non-parametrical values. Significant p-values are highlighted in bold.

## Discussion

This article formulates representative norm values including the associated TR and CI for the upper body posture of healthy male subjects aged 41 to 50 years. With regard to posture, it can be noted that the vertebral, shoulder and pelvic geometry of the test subjects is relatively harmonious, except for thoracic kyphosis, since the inclinations and rotations mentioned do not deviate by more than 4 mm or 4° with respect to a vertical perpendicular. There is a moderate ventral inclination in the sagittal plane, a minimal left-lateral inclination in the frontal plane and a rotation of the spine to the right. The thoracic bending angle is about 5° more pronounced than the lumbar bending angle. The kyphosis angle of 51.08° is also much larger than the lordosis angle of 32.86°, which illustrates the findings of the more pronounced kyphosis. The left shoulder, like the left pelvis, is slightly higher than on the right side. These minor deviations could be due to the fact that only healthy volunteers without postural complaints were included in the study. Representative norm values for the upper body statics near the symmetry axis and 0° axis for healthy men between 41 and 50 years of age can thus be determined.

Since, in addition to the BMI, the body height has also shown a significant correlation with the trunk length, but not the body weight, it can be concluded on the basis of the already existing results that taller people have a lower BMI than smaller ones. However, it should be considered that the standard deviation of the body height is ±7.29 cm and that of the BMI ± 3.96 kg/m². Nevertheless, this potential finding should be analysed more comprehensively in further studies.

When comparing these results with those of Ohlendorf *et al*.^20^ on male volunteers aged 18 to 35 years, it is noticeable that the participants in the present study displayed an overall higher body weight (by 9.0 kg) and a higher BMI (by 2.9 kg/m^2^). The body heights are comparable with a small difference of 2.0 cm. The results of the two studies are almost completely consistent with the data provided by the Federal Statistical Office^27^ for the respective age groups. In addition, in comparison with the age- and gender-specific results of the representative study by Mensik *et al*.^28^ on 7116 participants, no constitutional value deviated by more than 2.0 cm, 2.0 kg or 2.0 kg/m^2^. In old age, due to the physiological decrease in muscle mass and the resulting reduction in basal metabolism, the body’s overall energy consumption decreases^29^. As a result, body weight and BMI tend to increase which could explain the findings at hand. The comparison of the present data with a total of 102 healthy male volunteers aged 18–35 years^20^ regarding norm values shows that most of parameters of the posture tended to be almost identical. For example, the authors analyzed a sagittal trunk inclination moderate (approx. 4.0°) to the ventral, an axial deviation minimal to the left lateral and a discrepancy between thoracic kyphosis and lumbar lordosis. The discrepancies in the values of the kyphosis angle (45.85°) and lordosis angle (30.67°) are largely congruent in their tendency with the findings of the present study. While the lordosis angle of the 41–50 year-old volunteers at 32.86° largely corresponds to the value determined by Ohlendorf *et al*.^20^, there is a larger discrepancy with regard to the kyphosis angle at 5.23°. A possible explanation for this circumstance could be the increasing kyphosis in old age. A degenerative change and narrowing of the intervertebral discs in the anterior region increases the physiological thoracic kyphosis^30,31^.

Furthermore, the shoulder parameters also largely correspond to the findings of the present study^20^. However, the shoulder distance of 18–35 year olds is approximately 7 mm smaller, which could possibly be due to the smaller BMI mean value of 21.76 kg/m^2^ compared with the present study - or to the smaller body height (on average by 2.0 cm). The left shoulder blade is more caudal than the right and the right shoulder is slightly dorsally rotated. In addition, the right shoulder stance angle is 3.0° greater than the left, indicating a more caudal position of the right shoulder. All these findings of Ohlendorf *et al*.^20^ show, with the exception of the kyphosis angle and the scapular distance, that they are largely congruent with those of the present study. The same is true for the pelvic parameters where no discrepancy of more than 1° or 1 mm results when looking at the values.

Gender- and age-specific differences can be investigated by comparison with another study by Ohlendorf *et al*.^22^ on 106 female volunteers aged between 21 and 30 years using the same measurement system as in the present study. While most parameters (e.g. trunk inclinations, rotations of the spine, shoulder parameters) tend to be congruent, the pelvic distance differs anatomically by about 7.0 mm and the shoulder distance by about 36.0 mm. A large difference exists in the ratio between the respective extent of the kyphosis and lordosis angle. While the discrepancy between the two angles is approximately 5° for women, it was found to be approximately 18.0° for men in this study. It is noticeable that the lordosis angle in women (46.21°) is considerably larger than in men (32.86°); this divergence could be due to morphological differences. The pelvic blades and, consequently, the pubic bone angle are larger in women^32^. Furthermore, a larger pelvic tilt angle of 60.0° is given for women when compared with men, having an angle of 50.0–55.0°, so that the pelvis of women is more inclined overall and, consequently, the lordosis angle is larger than in men^29^. There are, therefore, age- and gender-specific differences with regard to the standard values.

However, further scientific extensions of other age decades of both sexes should be made to confirm the data obtained. Furthermore, the BMI should also be considered as an influencing factor on postural control in further analyses. In particular, the BMI should be taken into account as an influencing factor on postural control, especially in view of the fact that the BMI is increasing on average, especially in the industrial nations. Also in this study 65% of the participants have a BMI > 25 kg/cm². These differences might be even slightly larger, since anatomical correct marker placement in obese subjects proved to be difficult.

As a further aspect in future studies, the influence of work behaviour could be taken into account, i.e. whether the occupation is executed predominantly in a seated or standing position. In the context of this study it was only noted that 58% of the subjects have an office job, but not how many hours per day are spent sitting or standing or moving and to what extent this fact could influence the upper body posture. Possible sources of measurement error for future studies should also be taken into consideration. These include, for example, possible system errors in the equipment used or the placement of markers in the anatomically correct position^16,17^. Since the marker placement was always carried out according to the same scheme and the measurements were carried out by experienced scientists, the markers could also be placed adequately in obese people. Hairy volunteers had to be shaved at the marker sites. The only problematics of marker recognition were many dark or colorful tattoos on the skin areas where the markers were placed or a very pronounced cervical spine kyphosis at C7.

## Conclusion

With the help of video raster stereography, representative norm values for the upper body posture of healthy men between the ages of 41 and 50 years could be formulated. Their posture was close to the axis of symmetry. Overall, a low ventral inclination with a negligibly low left lateral axial deviation was observed. The left shoulder and the left pelvis were about 1 mm higher compared to the right side. Due to a higher BMI (in particular due to a higher weight), the shoulder blade distance is greater than that of the subjects aged 18 to 35 years. Men in the present age group (41 to 50 years) also show a kyphosis angle that is approximately 5.0° greater. The determined values can be used as reference values for future studies as well as for clinical routines.

## Data Availability

All relevant data are in the manuscript.
